# Acute Hemorrhagic Edema of Infancy after Coronavirus Infection with Recurrent Rash

**DOI:** 10.1155/2017/5637503

**Published:** 2017-01-24

**Authors:** Hannah Chesser, Jeffrey M. Chambliss, Eric Zwemer

**Affiliations:** ^1^Department of Pediatrics, University of North Carolina, Chapel Hill, NC, USA; ^2^Department of Pediatrics, University of Texas Medical Branch, Galveston, TX, USA

## Abstract

Purpura, particularly when accompanied by fever, is a worrisome finding in children. Acute hemorrhagic edema of infancy (AHEI) is a benign type of small-vessel leukocytoclastic vasculitis that presents with progressive purpura and has an excellent prognosis. Patients with AHEI present with large, target-like purpuric plaques affecting the face, ear lobes, and extremities. While the rapid onset of these skin findings can be dramatic, the child with AHEI is usually well appearing with reassuring laboratory testing. We describe a case of a previously healthy 8-month-old female who presented with progressive purpura in a nondependent distribution, low-grade fevers, and extremity swelling. An extensive workup was performed prior to making the diagnosis of AHEI. Coronavirus was implicated as the likely triggering pathogen, and the patient suffered a recurrence of purpuric rash and swelling several weeks after her initial presentation.

## 1. Introduction

Purpura with fever is a worrisome finding in children, raising the possibility of meningococcemia, disseminated intravascular coagulation, or drug eruption [1]. A common cause of purpura among pediatric patients is Henoch-Schönlein Purpura (HSP), with the classic presentation of dependent purpura, renal disease, abdominal pain, and arthritis or arthralgias. Acute hemorrhagic edema of infancy (AHEI), however, is a less common etiology of pediatric purpura with approximately 500 reports in the literature. AHEI presents with purpuric lesions of the face, ears, and extremities, and nonpitting edema of the extremities. Although the lesions have a dramatic onset over a 24- to 48-hour period, the child with AHEI is nontoxic appearing without visceral involvement [2]. Patients with AHEI usually make a complete recovery within 1–3 weeks of presentation with supportive care only [3]. Recurrence of symptoms is rare, described in only three other published reports to our knowledge [4].

We describe a case of an 8-month-old female who presented with progressive purpura in a nondependent distribution, low-grade fevers, and extremity swelling and who was ultimately diagnosed with AHEI. To our knowledge, this is the first case of AHEI associated with coronavirus* NL63* and one of the first to demonstrate recurrence.

## 2. Case Presentation

An 8-month-old previously healthy female was admitted for evaluation of progressive purpura and extremity swelling. The rash initially began on her inner thighs and rapidly progressed over the course of the day to the soles of her feet, face, and bilateral ears. She also developed swelling of her hands, feet, and right eyelid. Despite the dramatic progression of her rash, she remained happy and playful with normal oral intake.

Her acute symptoms were preceded by a mild cough for one week and two days of bilateral conjunctivitis with clear, mucoid discharge. Review of systems was otherwise negative for diarrhea, bloody stools, abdominal pain, vomiting, gross hematuria, change in urination, or joint swelling or pain.

**Figure 1:**
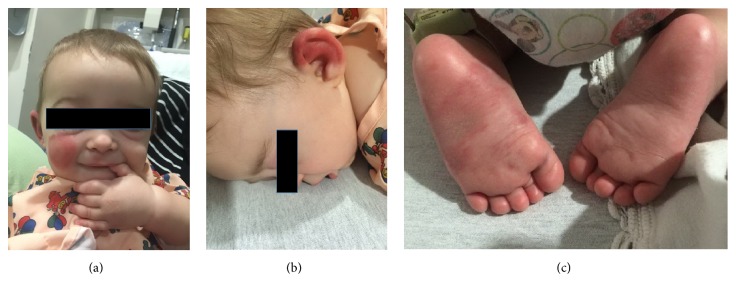
Exam findings. (a) Purpura on right cheek and hand edema. (b) Purpura of ear. (c) Violaceous nonblanching plaques on feet.

Vital signs were notable for a fever of 38.5°C and a normal blood pressure of 100/59. Physical exam showed a smiling infant with multiple erythematous and violaceous nonblanching plaques over her face, trunk, feet, and bilateral ears. Nonpitting edema of her hands and feet was also appreciated (Figure 1).

Laboratory testing showed white blood cell count 11,6000 per microliter, platelets 437,000 per microliter, blood urea nitrogen 6 mg/dL, creatinine 0.23 mg/dL, prothrombin time 10.6 sec, and activated partial thromboplastin time 33.7 seconds. Urinalysis was also normal. C-reactive protein was mildly elevated at 3.3 mg/dL (reference range 0.0–1.0 mg/dL).

She was initially started on intravenous ceftriaxone with concern for possible bacteremia. Overnight, her extremity swelling worsened and she developed new purpuric lesions, though remained well appearing. The diagnosis of AHEI was made the following morning based on clinical characteristics and in consultation with a dermatologist and a rheumatologist. No skin biopsy was performed given the classic appearance of the rash. Antibiotics were discontinued. A respiratory viral panel sent on admission returned positive for coronavirus* NL63* by nucleic acid amplification testing.

48 hours after the onset of purpura, her rash began to dissipate and fade along with the edema. Corticosteroids were not administered due to this clinical improvement. She had complete resolution of her symptoms three days later.

Three weeks after initial presentation, the patient had a recurrence of periorbital and extremity swelling and purpuric rash without end organ involvement. She had resolution of these symptoms within four days with supportive care only.

## 3. Discussion

AHEI is a small-vessel leukocytoclastic vasculitis that causes benign purpura in children typically between the ages of 4 and 24 months [5]. It classically presents with rapidly progressive purpuric lesions over the face, extremities, and bilateral ears, accompanied by nonpitting edema of the extremities. The presence of bilateral auricular swelling and purpura in a well-appearing child should raise particular clinical suspicion for AHEI. Fewer than 10% of patients diagnosed with AHEI exhibit extracutaneous manifestations, which include glomerulonephritis, abdominal pain, arthralgia, testicular torsion, and intussusception [6]. Diagnosis is clinical and can be made without a skin biopsy. If skin biopsy is performed, a leukocytoclastic vasculitis is present with IgA immunofluorescence in approximately one-third of patients [6]. Laboratory testing is typically reassuring without evidence of renal or hematologic compromise. Some physicians have made the diagnosis via telemedicine, relying on cellphone photos to monitor the progression of the disease [7].

About 75% of cases of AHEI are preceded by respiratory infections, diarrheal illnesses, or urinary tract infections. Viruses including rotavirus, herpes simplex virus, and adenovirus have been implicated [3]. Additionally, antibiotics and vaccinations have been identified as triggers [8]. Although the exact triggering pathogen for AHEI is rarely identified, our case demonstrates that coronavirus can precede this disease. Our patient's symptoms of cough and conjunctivitis are consistent with coronavirus infection, and the result is unlikely to be a false positive given the low rate of coronavirus detection observed in healthy children [9]. Specifically, coronavirus* NL63* has been associated with petechial rash, but this is the first case to our knowledge to be associated with AHEI [10].

Several case reports note rapid improvement of purpura or edema after the administration of corticosteroids [3, 11]. Given the improvement in our patient's purpura by 48 hours after onset, we made the decision not to administer corticosteroids. Our case illustrates that symptoms of AHEI can have rapid onset as well as swift resolution without steroid intervention. This suggests that some previously reported patients may have improved even without corticosteroids, though at least one case has noted relapse of symptoms when steroids were removed [7]. While use of corticosteroids remains controversial, most reports suggest that corticosteroids only be considered in severe presentations with complications or inability to maintain fluid intake [7, 11, 12].

With or without corticosteroids, most patients with AHEI make a complete recovery within one to three weeks of presentation [3]. To our knowledge, our case is the fourth report in which a recurrence of symptoms occurred [4]. In most cases, the recurrence occurred in a three-week time frame from onset of symptoms, though one case series reported a familial occurrence of AHEI in which three sons have had frequent relapses of purpuric circular rash into adulthood [13].

This patient was admitted to the hospital, underwent significant laboratory workup, received intravenous antibiotics, and was evaluated by multiple subspecialty services prior to diagnosis. While keeping in mind more worrisome diagnoses, providers should consider the diagnosis of AHEI in well-appearing young children with purpuric lesions on the face and ears and nonpitting edema of the extremities. Awareness and early recognition of AHEI may prevent hospital admission, invasive workup, and parental and provider concern.
